# Genome-wide diversity, linkage disequilibrium, and admixture in the main Colombian Creole pig breeds

**DOI:** 10.1007/s11250-024-04140-6

**Published:** 2024-10-10

**Authors:** Ricardo José Ocampo-Gallego, Yolanda Gómez Vargas, Juan Carlos Rincón Flórez

**Affiliations:** 1 Corporación Colombiana de Investigación Agropecuaria - Agrosavia. Nus Research Center, Corregimiento San José del Nus, San Roque, CP. 250047 Antioquia Colombia; 2 Palmira Zoogenetic Resources Research Group, Deparment of Animal Science, Universidad Nacional de Colombia, Palmira, Valle del Cauca, Colombia

**Keywords:** American swine, Animal genetic resources, Indigenous pigs, Population structure, South American pigs

## Abstract

Colombian Creole pigs have adapted to tropical conditions for over 500 years. They have been modified by natural and artificial selection in different regions. At present, the diversity and current introgression status are unknown. The objective was to estimate the genomic diversity, linkage disequilibrium, population structure, and admixture of four Colombian pig breeds and their relationship with other breeds worldwide. Three Colombian pig breeds (SPE-San Pedreño, 11 samples; ZUN-Zungo, 11 samples; CM-Casco de Mula, ten samples) from the conservation nucleus and one biotype not recognized as a breed (CCH-Criollo Chocoano, seven samples) were genotyped using the Illumina GGP-Porcine80K chip. Open-access data from seven international breeds were also included. Colombian Creole pigs showed moderate genetic differentiation (F_ST_ 0.14) globally, but several groups of animals separated, suggesting local clustering due to geographical isolation or different founding effects. Colombian Creole pigs showed breed imprinting and specific grouping in all analyses except for CCH, which, like the Ecuadorian Creole, was a cluster of admixtures. The Colombian Creole pigs revealed a significant relationship with the Iberian pig and some other breeds to varying degrees. However, good maintenance of the conservation nucleus was evidenced. Potential adaptive genes, mainly related to immunological functions, were found, according to F_ST_ and pcadapt analyses. This study provides a foundation and scientific data for policy decisions on zoogenetic resources.

**Supplementary Information:**

The online version contains supplementary material available at 10.1007/s11250-024-04140-6.

## Introduction

The domestication of livestock is one of the most critical events in human history. It has significantly impacted human life, enabling the transition from hunting and gathering to a more stable lifestyle. Pigs (*Sus scrofa*) are economically important animals domesticated in much of East Asia and China about 10,000 years ago (Larson et al. 2010). Since then, under various evolutionary forces such as migration, mutation, selection (natural and artificial), and genetic drift, they have given rise to numerous pig breeds with diverse phenotypes in appearance, growth, palatability, fertility, and other traits of economic interest (Huang et al. 2021).

Most indigenous livestock breeds are facing the challenge of modern commercial breeds, and this is causing animal genetic resources to decline at an alarming rate around the world; it is estimated that 20% of indigenous breeds are currently on the verge of extinction (Herrero-Medrano et al. 2013). In the case of Colombian pig genetic resources, the country has seven locally adapted biotypes, some of which are not recognized as breeds, such as the Criollo Chocoano pig, and only three that are formally recognized as Creole pig breeds and reported in the ’FAO’s Domestic Animal Diversity Information System ( Food and Agriculture Organization of the United Nations [FAO] 2019). The breeds recognized in Colombia are the San Pedreño in the Andean region, the Casco de Mula in the Orinoco region, and the Zungo in the Caribbean region (Ocampo and Abuabara 2021). These breeds are believed to have originated from the Iberian pig breed from Extremadura and Andalusia in Spain (Sánchez et al. 2015). According to Benítez Ortiz and Sánchez (2001), the biotypes that contributed the most genes to the Creole pig were the Negra Lampiña and Negra Entrepelada, although no research has proven this.

The Colombian Creole pig breeds represent a biodiversity heritage because they belong to a very heterogeneous population, the product of crossbreeding and adaptation of the first Iberian breeds. They have naturally survived and adapted to different agroecological conditions, infectious factors, and nutritional constraints, becoming a reservoir of genetic variability that enriches and refreshes commercial germplasm. They are also a source of food and income for many small subsistence farmers in the country (Barrera et al. 2007).

Despite their hardiness and remarkable ability to adapt to unfavorable environmental conditions, the Colombian Creole pig is endangered. In the last century, improved foreign breeds with white coats have attracted more interest from pig farmers due to their better production yields and lower fat carcasses, leaving the breeding of Creole pigs in the hands of small-scale farmers (Ocampo et al. 2019). In Colombia, however, Creole breeds have become increasingly important due to their fertility, adaptability, and resistance to disease, which has sparked an interest in conserving and documenting animal genetic resources and resulted in several studies aimed at characterizing and documenting the genetic diversity and productive component of different domestic species of economic interest, including pigs (Ocampo et al. 2017).

The SNPs (single nucleotide polymorphism) markers distributed in the genome have been widely used in studies to assess genetic diversity, population structure, inbreeding level, and admixture and detect selection fingerprints in cattle (Bejarano et al. 2018; Ocampo et al. 2021) and pigs (Muñoz et al. 2019; Wang et al. 2019; Huang et al. 2021), either by genotyping by sequencing or by genotyping arrays. However, the quantification of genetic diversity, population structure, and genomic inbreeding in Colombian Creole breeds using genome-wide data has been little studied, and little is known about the diversity, population structure, and admixture they have undergone. Therefore, this work aims to increase the knowledge of genomic diversity, linkage disequilibrium, population structure, and admixture of four Colombian pig breeds and their relationship with other species worldwide using genome-wide SNP markers.

## Materials and methods

### Sampling and genotyping

Colombian pig breeds are in danger of extinction, with very few purebred representatives (Table 1), making it difficult to obtain samples from unrelated individuals. For this study, the main Colombian Creole pig breeds were considered, selecting representative individuals of the species and avoiding highly related animals (parent-offspring, full siblings, half siblings and grandparent-grandchild). We included samples of 11 animals of the San Pedreño breed (SPE), 11 of the Zungo breed (ZUN), and 10 of the Casco de Mula breed (CM) from Agrosavia’s germplasm bank of the Nation for Food and Agriculture, the custodian of this genetic resource in Colombia. In addition, seven samples of Criollos Chocoanos (CCH), kept by local people in semi-wild conditions in the jungle areas of Choco, were included. Blood samples were collected from the jugular vein using BD Vacutainer tubes containing k3-EDTA for DNA collection. The samples were transported to ’Agrosavia’s molecular genetics laboratory, where leukocyte DNA was extracted using ’Qiagen’s commercial genomic DNA extraction kit (Dneasy Blood and Tissue kit^®^). The samples from the CCH pigs were collected similarly and DNA was extracted using the same kit but at the Molecular Biology Laboratory of the Universidad Nacional de Colombia, Palmira, according to the ’manufacturer’s specifications.

### Genotyping

Thirty-nine Colombian Creole pigs were genotyped and divided into four groups, including the Criollo Chocoano biotype, which is not yet recognized as a breed (Table 1). The animals were genotyped using the Illumina GGP Porcine80K chip (70,208 SNPs per animal) at ’Agrosavia’s molecular genetics laboratory. INDELS, SNPs in sex, and mitochondrial chromosomes were removed in the initial processing using R software (R Core Team 2022). After editing, 62,331 autosomal SNPs were available, with a call rate of 0.93. From the database, quality control was performed by retaining genotypes and then individuals with call rate > 0.95, following FAO’s pipeline for quality control of genomic data (Ajmone-Marsan et al. 2023). After quality control, there were 55,432 SNPs in 18 autosomes and 37 individuals, with a call rate of 0.99 per SNP and individual.

**Table 1:** Breeds, sample origin, population, and number of animals sampled in this study

Breed	Sample Origin	Population (DAD-IS 2019)	*N* (*N* after QC)	Source
Ibérico (IBE)	Spain	737,419	16 (15)	Burgos-Paz et al. (2013)
Canaria (CA)	Spain	1,739	5 (4)	Burgos-Paz et al. (2013)
Bisaro (BIS)	Portugal	45,890	16 (14)	Burgos-Paz et al. (2013)
Landrace (LAN)	USA	44,036	20 (20)	Burgos-Paz et al. (2013)
Meishan (MEI)	China	No data	17 (17)	Burgos-Paz et al. (2013)
Criollo de Ecuador (EC)	Ecuador	No data	5 (4)	Burgos-Paz et al. (2013)
Zungo COL (ZCO)	Colombia	128	10 (10)	Burgos-Paz et al. (2013)
Criollo Chocoano (CCH)	Colombia	No data	7 (5)	This paper
Casco de Mula (CM)	Colombia	138	10 (10)	This paper
Zungo (ZUN)	Colombia	128	11 (11)	This Paper
San Pedreño (SPE)	Colombia	99	11 (11)	This paper
Total			128 (121)	

### Linkage disequilibrium (LD) and effective population size (ne)

From the above database, the post-editing MAF was estimated for each Colombian Creole breed using PLINK v1.90 (--freq) software. The LD r^2^ for each breed was calculated between all pairs of adjacent SNPs for each chromosome at 0 to 1 Mb using PLINK v1.90 (--ld-window-r2) (Chang et al. 2015). Moreover, the results were analyzed and plotted against distance to observe the LD decay up to a length of 1 Mb for each Colombian breed using the ggplot2 package of R (R Core Team 2022). The .ped files were then used to estimate the Colombian Ne using SneP v1.1 software based on the relationship between LD and recombination rate, according to Corbin et al.‘s (2012) equation, assuming that 1 Mb = 1 cM. The Ne was estimated for 50 generations using the files implemented in the LD analysis.

### ROH analysis

From the above database (without quality control by MAF), Runs of Homozygosity (ROH) were identified for each breed using PLINK v1.90 (--homozyg and –homozyg-group options) and a sliding window approach with 50 SNPs, an overlap ratio of 0.05, a minimum length to declare a homozygous segment of 1000 kb, and a density of 1 SNP per 150 kb, containing at least 20 SNPs. According to Ferenčaković et al. (2013), no heterozygous markers were allowed. ROHs were grouped into segments of 1–4 Mb, 4–8 Mb, 8–12 Mb, and > 12 Mb. From these measurements, inbreeding per ROH (FROH) was estimated according to Bjelland et al. (2013), considering an autosomal genome length of 2,265,770 kb (Sscrofa11.1: GCA_000003025.6/). The length of the autozygous segments was assumed to follow an exponential distribution with mean t = ½g Morgans, where g is the number of generations since a common ancestor (Howrigan et al. 2011), which, in the case of segments larger than 12 Mb, would indicate the last 4.17 generations.

### Diversity analysis

The previous database was pruned using the --indep-pairwise 50 10 0.2 option of PLINK v1.90, which develops the paired LD estimation in windows of 50 SNPs, considering 10 SNPs to change the window and a threshold of r^2^ = 0.20. After pruning, 7,256 SNPs were counted with an average call rate of 0.99. From the database, observed and expected homozygosity (Ho and He), MAF, and homozygote excess inbreeding (FHOM) were calculated according to Keller et al.‘s (2011) equation. Population structure was also estimated by F_ST_ per marker and averaging using.

### Multibreed analysis

The number of individuals for each breed is shown in Table 1. The Iberian, Canary Island, and Bisaro pigs were included to establish ancestral relationships due to a common origin before arriving in the Americas. We included the Ecuadorian Creole (EC) because of its most recent evolutionary relationship and geographical proximity, the Zungo from Colombia as a control for merging the databases, the Landrace for the possible introgression of the commercial lines used in Colombia, and Meishan as an outgroup. The information obtained from the Illumina 60 K chip consisted of 46,259 genotypes of autosomal SNPs, which were merged with the raw database of Colombian Creoles using the --bmerge and --flip options of PLINK v1.90, using forward coding in both cases, checking for correct genotypic correspondence between the databases. In addition, a common breed in the databases was used as a merging control. The databases presented 33,564 common autosomal SNPs, which, after the quality control described above (call rate per SNP and individual > 0.95, MAF > 0.05), allowed us to have 33,299 SNPs and 121 individuals (call rate 0.99 per SNP and per individual).

### Population structure and admixture

The merged database was pruned using the --indep-pairwise 50 10 0.2 option of PLINK v1.90, as described above. This resulted in 7,070 SNPs and 121 individuals (call rate 0.99). With this database, principal component analysis (PCA) was performed using the --pca command, F_ST_s were estimated using the --fst function, and genomic distances from the first to the fifth nearest neighbor were evaluated using the --neighbor function of PLINK v1.90. PCA plotting was performed using R’s ‘GGPLOT2’ package (R Core Team 2022). Subsequently, the genomic IBS matrix was constructed using the “SNPSTATS” package of R (Clayton 2020). Also, Nei population genetic distances were estimated with the “ADEGENET” package (Jombart 2008), and an NJ phylogenetic tree representation was constructed using the “APE” package (Paradis and Schliep 2019) and plotted using the “FACTOEXTRA” package (Kassambara and Mundt 2020).

In parallel, the .ped files were converted to Arlequin files using PGDSpider 2.1 software (Lischer and Excoffier 2012). Each population was defined according to breed, and Slatkin’s paired F_ST_ statistics were estimated using Arlequin software (Arlecore for Linux) (Excoffier and Lischer 2010). Gene flow, expressed as the number of migrants (Nm) per generation, was calculated using the following expression derived from Wright’s statistics: Nm = [(1/F_ST_) − 1]/4.

To detect population structure based on Nei genetic distances, we used the “NETVIEWER” package (Steinig et al. 2016), which relies on an unsupervised clustering method known as superparamagnetic clustering (Spc). The “plot_kselect” function evaluates the optimized value for the maximum number of nearest neighbors (K-NN) an individual can have, with values ranging from 1 to 20, exploring the structure from the fine to the larger scale. The population networks were then visualized using different node colors for each breed.

Treemix analysis was performed (Pickrell and Pritchard 2012), which infers splitting and mixing patterns from genome-wide frequency data, allowing the maximum likelihood tree for a group of populations and the number of admixture events to be observed. This was done by estimating the stratified frequencies (--write-cluster and --freq) from the .ped files and creating the Treemix file with the plink2treemix.py script. From this, an ML tree was constructed. Bootstrap replicates were generated to define the tree topology through contiguous SNP blocks of 500 SNPs (-k 500). The Treemix algorithm computes the number of migration edges in a population tree. In order to define the optimal number of migrants, migration edges are usually added when 99.8% of the variation in the data is explained. The R package ‘OPTM’ was used to automate this process (m = 1–10), allowing the optimal number of migrants to be defined, using the linear method. The graphical representation of the tree with mixing events was generated by R (R Core Team 2022) using the “plotting_funcs” R script of the Treemix software (Pickrell and Pritchard 2012).

An analysis was then performed with ADMIXTURE (Alexander et al. 2009) to identify admixture events and genetic composition of the Colombian Creole pigs and evaluate the breed composition. For this, k from 2 to 15 possible ancestral populations were considered, and cross-validation error analysis was performed to select the best k through the R package “Starmie” (Tonkin-Hill and Stuart 2021). From this information, the k from 2 to 10 possible ancestral populations were plotted with R (R Core Team 2022).

Finally, the R package “pcadapt” detected genetic regions related to biological adaptation processes; the library provides statistical tools for outlier detection based on the population structuring found in the PCA. From the *p*-values found for the pcadapt analysis markers (with k = 3) and the PLINK F_ST_ values, a circular Manhattan plot was made using the R package “CMplot” (Yin et al. 2021). The threshold was defined according to the Benjamini-Hochberg correction for the pcadapt analysis (α = 0.05); for F_ST_, the threshold was defined at F_ST_ >0.5, indicating a high genetic differentiation. Considering the persistence of LD in pigs, an interval of 500 kbs upstream and downstream of the detected marker was considered for candidate genes. The search for genes and QTLs around candidate markers was performed with the R package “gallo” (Fonseca et al. 2020; R Core Team 2022), and the annotation was performed through the functional annotation tool DAVID (Sherman et al. 2022). Using the REVIGO web server, a summary was made of the list of Gene Ontology (GO) terms related to biological processes (allowing a semantic similarity of 0.7).

## Results

### Diversity, linkage disequilibrium (LD), and effective population size (ne)

**Fig. 1:**
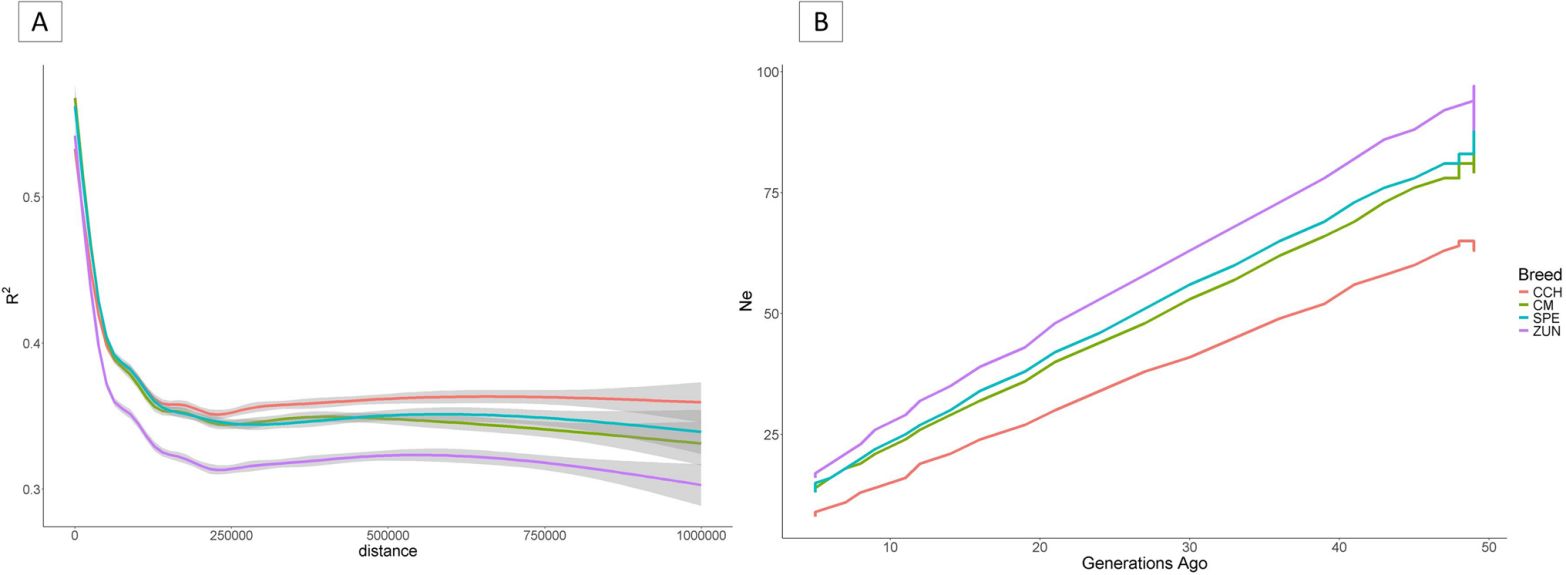
**A** Linkage disequilibrium decay and (**B**) Effective population size by generation in colombian creole pig breeds

The MAF distribution found was negative skew, with most markers having intermediate frequencies. Thus, 2.64% of the autosomal SNPs were found to be fixed among the Colombian creole pigs. 6.28% had MAF less than 0.05, 13.25% less than 0.1 and 30.43% less than 0.2. After the initial quality control of the genotypes (before pruning) for each Colombian breed (55,432 SNPs), we found an MAF ranging between 0.237 (CM and ZUN) and 0.276 (CCH). This suggests a higher number of alleles that segregate in CCH (refer to Table 2). The average LD of the SNPs analyzed ranged from 0.342 at an average distance of 207.20 kb in ZUN to 0.375 at an average distance of 207.40 kb in CCH. This implies that the LD in the CCH pig is more persistent, as reflected in the LD decay graph shown in Fig. 1A. In this graph, CCH displays a higher degree of LD persistence, followed by CM and SPE with similar and intermediate traces, and finally, a lower persistence in the ZUN breed. A trend related to Fig. 1B shows a decrease in Ne of Colombian Creole pig breeds in the last 50 generations. Generally, all evaluated breeds show a decrease in Ne, with CCH having the lowest current Ne at 10 and ZUN having the highest at 19, as presented in Table 2.

After pruning the genotypes, the average F_ST_ was 0.125. An MAF ranging from 0.287 (CM) to 0.321 (CCH) was detected. The lowest observed homozygosity (Ho) was found in CCH at 0.381, while the highest was in SPE and ZUN at 0.421. However, the lowest expected value was 0.358 (CM), while the highest was CCH at 0.399. CCH was the only population with a deficit of heterozygotes (F_IS_ = 0.093). All other breed groups had an excess of heterozygotes associated with a tendency to exogamy, with values ranging from − 0.115 in SPE to -0.175 in CM (Table 2).

**Table 2:** Diversity parameters in Colombian creole pig breeds using an illumina 80 K genotyping chip

Breed	Criollo Chocoano (CCH)	Casco de Mula (CM)	Zungo (ZUN)	San Pedreño (SPE)
Number of SNPs	55,432	55,432	55,432	55,432
MAF before pruning	0.276	0.237	0.237	0.241
Mean LD	0.375	0.371	0.342	0.372
Mean distance (kb)	207.40	206.35	207.20	206.21
Distance LD > 0.3 (kb)	200.68	197.37	197.17	197.5
Effective population Size	10	16	19	16
Number of SNPs pruned	7,256	7,256	7,256	7,256
MAF after pruning	0.321	0.287	0.293	0.291
Observed homozygosity	0.381	0.421	0.421	0.405
Expected homozygosity	0.399	0.358	0.364	0.363
F _IS_	0.093	-0.175	-0.153	-0.115

High levels of autozygosity were observed through ROHs, as shown in Table 3. The ZUN breed showed the lowest ROH value (9.97% ± 1.42), while the SPE breed had the highest (14.93% ± 2.33). Within the CCH breed, the most pronounced proportion of homozygosity was due to long fragments (> 12 Mb), indicating recent inbreeding, supported by the FIS and the heterozygote deficit mentioned earlier. In contrast, the CM, ZUN, and SPE breeds showed higher autozygosity for medium and small fragments (< 8 Mb), indicating older inbreeding. These results align with the obtained FIS values, which suggest a tendency toward exogamy.

**Table 3:** Inbreeding coefficient (%) distribution described by homozygosity (F_ROH_) runs in Colombian creole pig breeds from the 80k illumina chip analysis

Breed	Total F_ROH_	F_ROH_ (1 < 4 Mb) Old	F_ROH_ (4–8 Mb)	F_ROH_ (8–12 Mb)	F_ROH_ (> 12 Mb) Recent
Criollo Chocoano (CCH)	11.32 ± 3.23%	2.68 ± 0.91%	3.27 ± 1.01%	1.57 ± 0.47%	3.80 ± 1.06%
Casco de Mula (CM)	10.99 ± 1.65%	3.59 ± 0.39%	3.61 ± 0.75%	1.75 ± 0.32%	2.04 ± 0.49%
Zungo (ZUN)	9.97 ± 1.42%	3.31 ± 0.37%	3.04 ± 0.48%	1.50 ± 0.28%	2.12 ± 0.59%
San Pedreño (SPE)	14.93 ± 2.33%	5.18 ± 0.71%	4.30 ± 0.71%	2.36 ± 0.55%	3.09 ± 0.56%

(± standard deviation)

### Multibreed analysis

**Fig. 2:**
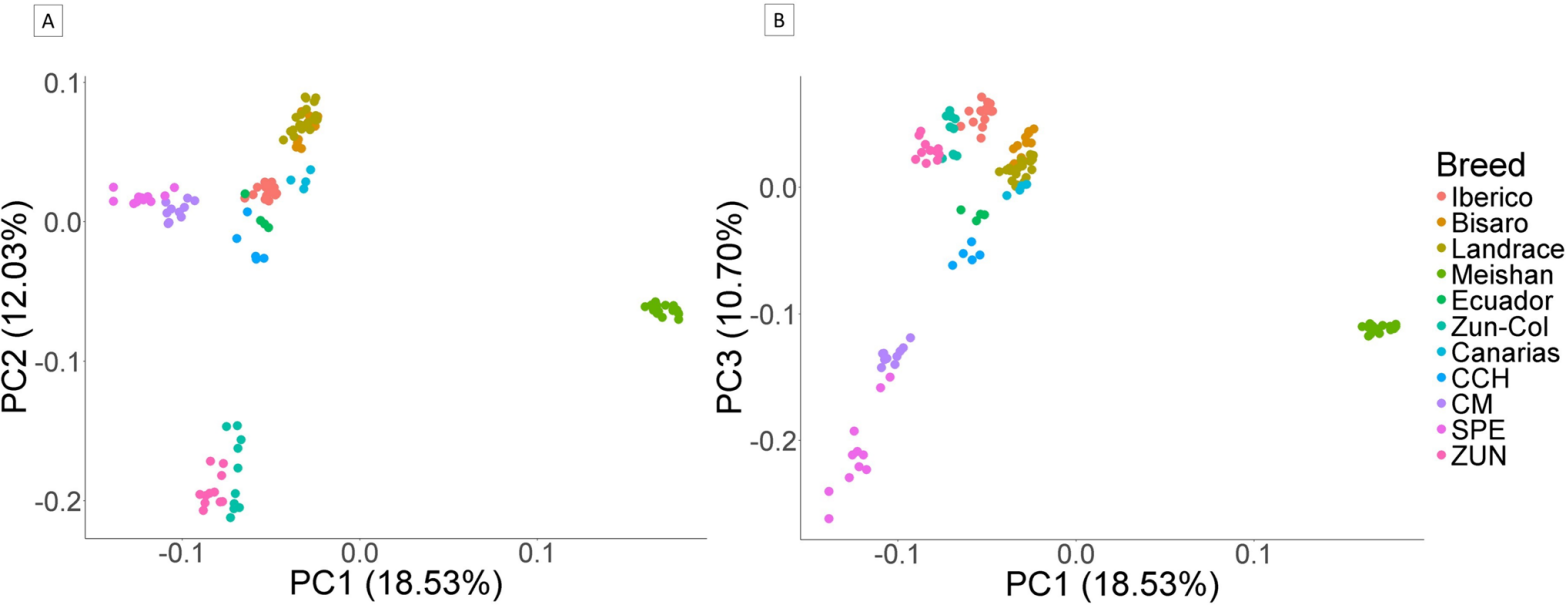
PCA plot displaying genetic relationships between Colombian creole pig breeds and other analyzed pig breeds. **A** PC1 vs. PC2 (**B**) PC1 vs. PC3

In total, 11 populations showed a mean F_ST_ of 0.182. Regarding PCA, the first component (PC1) explained 18.53% of the variance, PC2 12.03%, and PC3 10.70%. The PCA graph (Fig. 2.) shows that the databases were merged correctly because the ZUN pigs overlapped within a single cluster. The graph also shows that the first component separates Meishan (MEI) from the other breeds. The second component further separates ZUN pigs from other breeds, including Colombian Creoles. The third component separates CM and SPE from the other pigs. The graph reveals that CCH pigs are closely related to EC and CA pigs, with the Creole group being closer to the Iberian pig (IBE) and Landrace. Meanwhile, CM and SPE are relatively similar, and ZUN slightly differs from the other Colombian Creoles.

We evaluated the five nearest ancestors for all included breeds. We discovered that the individuals of each breed correspond to the five nearest ancestors of the same breed, except for the CCH pigs. Their closest relative was consistently of the same breed, but among the five closest ancestors were ZUN, CM, and EC individuals. This suggests a possible mixture or introgression in this genetic group.

**Fig. 3:**
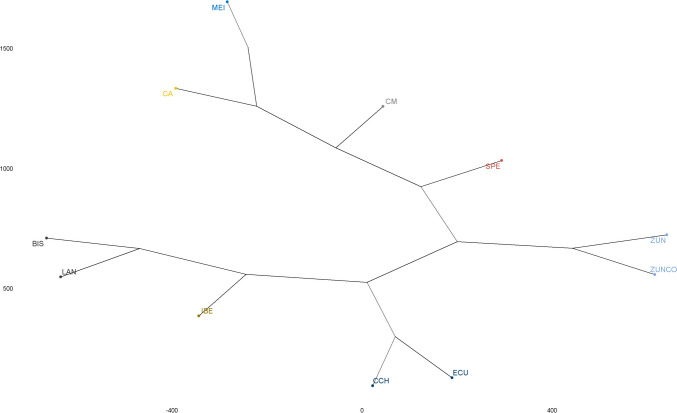
Neighbor-joining population tree from Nei genetic distances between the different pig breeds evaluated

Conversely, the neighbor-joining tree representing the Nei distances (Fig. 3) between populations revealed a closer proximity between the Colombian Creole pigs, illustrating a shared ancestry between the CCH and EC pigs. Similar to previous analyses, a shorter distance was noted between CM and SPE and a longer one concerning ZUN. This analysis also identified a greater proximity of IBE pigs to CCH and EC pigs.

As expected, the paired Slatkin’s F_ST_ showed the highest values between MEI and the other pig breeds (Table 4). When only looking at the Colombian pig breeds, there is a significant structuring between them, with the lowest value of 0.14 between the ZUN breed and the CCH and the highest Slatkin F_ST_ value between CM-ZUN (0.27) and CM-SPE (0.27). These values correspond to roughly 1.88 to 3.64 migrants per generation. When comparing pigs from Colombia with others, the CCH resulted into the lowest F_ST_ value (0.12) compared to the IBE and Landrace (0.09). Something similar can be observed in the EC, which not only has a short genetic distance from Iberian and LAN pigs but also displays weak genetic structuring from CCH (0.04 F_ST_), resulting in the highest number of potential immigrants among all pairs of F_ST_ (14.06 migrants per generation). These findings highlight a significant genetic relationship between these two pig groups based on their F_ST_ values.

**Table 4:** Pairwise Slatkin F_ST_ (below diagonal) and migrant numbers (above Diagonal) between various pig breeds evaluated, including Colombian creole breeds

Breed	IBE	BIS	LAN	MEI	EC	ZCO	CA	CCH	CM	SPE	ZUN
Ibérico (IBE)		3.42	3.32	1.18	5.41	2.60	2.11	4.14	2.21	2.21	2.50
Bisaro (BIS)	0.15		4.95	1.37	5.26	2.53	2.46	4.49	2.41	2.46	2.43
Landrace (LAN)	0.15	0.10		1.54	5.72	2.74	2.83	5.42	2.68	2.76	2.63
Meishan (MEI)	0.42	0.36	0.33		1.31	1.15	0.94	1.41	1.01	1.01	1.12
Criollo de Ecuador (EC)	0.09	0.10	0.09	0.38		3.54	2.90	14.06	3.34	3.15	3.45
Zungo COL (ZCO)	0.19	0.20	0.18	0.44	0.14		1.75	3.65	1.93	1.93	49.15
Canaria (CA)	0.24	0.20	0.18	0.53	0.17	0.29		2.73	1.61	1.68	1.70
Criollo Chocoano (CCH)	0.12	0.11	0.09	0.35	0.04	0.14	0.18		3.16	3.21	3.64
Casco de Mula (CM)	0.23	0.21	0.19	0.49	0.15	0.26	0.31	0.16		1.86	1.88
San Pedreño (SPE)	0.23	0.20	0.18	0.50	0.16	0.26	0.30	0.16	0.27		1.94
Zungo (ZUN)	0.20	0.21	0.19	0.45	0.14	0.01	0.29	0.14	0.27	0.26	

*All paired FST were statistically significant (p-value < 0.05), except ZCO vs. ZUN

**Fig. 4:**
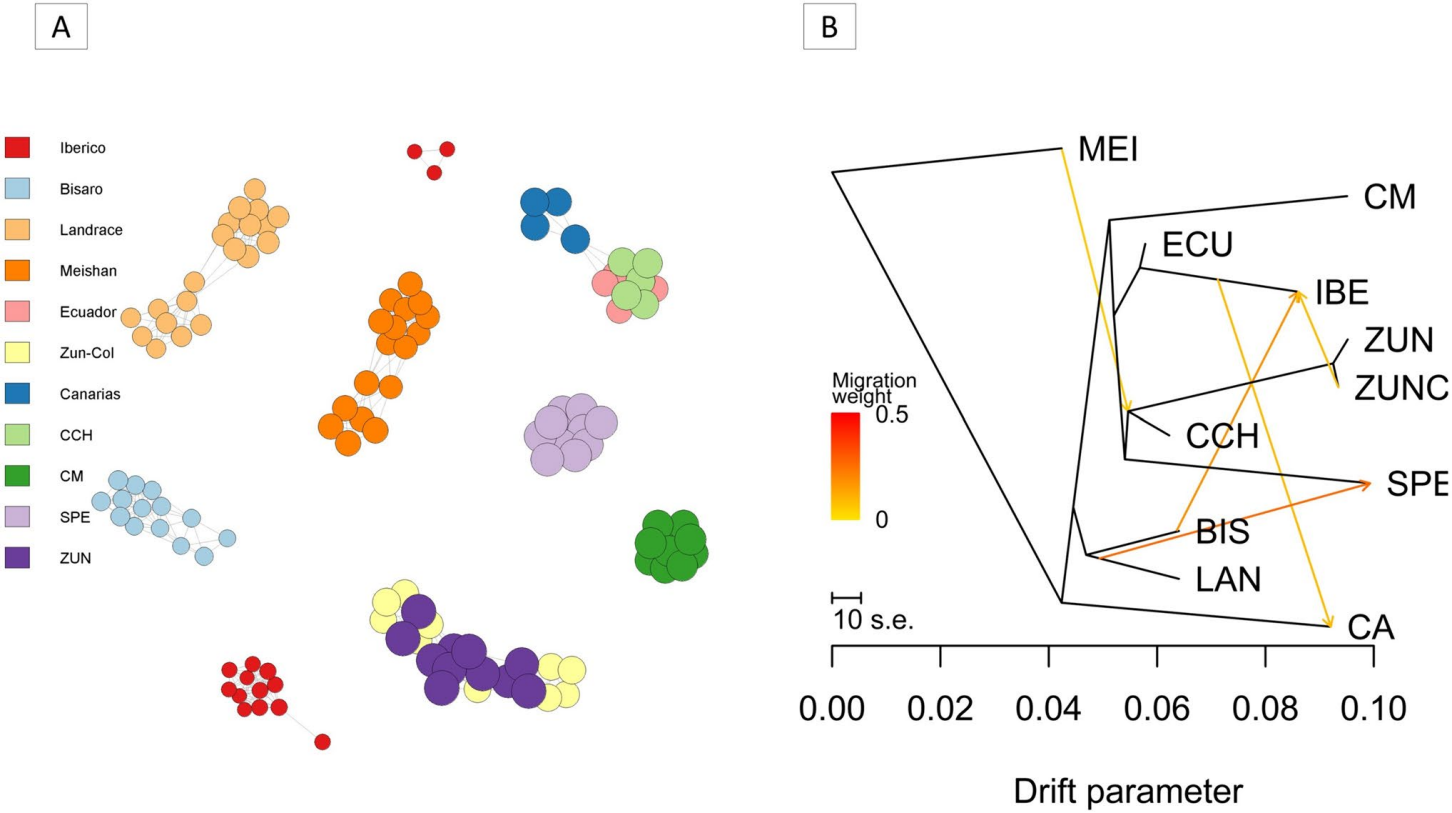
**A** Netview population structure based on Nei genetic distances between the different pig breeds evaluated. The optimized maximum number of nearest neighbors (K-NN) = 10. **B** Treemix representation of pig populations evaluated

On the other hand, the optimized maximum number of nearest neighbors (K-NN) was 10 (Supplementary Fig. 1A), so the Netview analysis (Fig. 4) was performed, showing the formation of groups according to each breed. Once more, a close relationship between CCH and EC emerges, and there is a higher correlation between CCH and the CA breed compared to the IBE.

The Treemix diagram displayed on the ML tree reveals a noticeable separation of MEI as an outgroup (see Fig. 4B). Moreover, there is a grouping amongst the Colombian pig breeds related to the EC and IBE pigs, creating a cluster. CM partially splits from the Colombian breeds. Regarding the most appropriate number of migration events was approximately m = 5 (see Supplementary Fig. 1B). The analysis shows a higher rate of migration for LAN into SPE. Additionally, there are introgression events of IBE into CA, BIS into IBE, ZUN into IBE, and MEI within the common branch CCH-ZUN, although at a lower rate (see Fig. 4B).

**Fig. 5:**
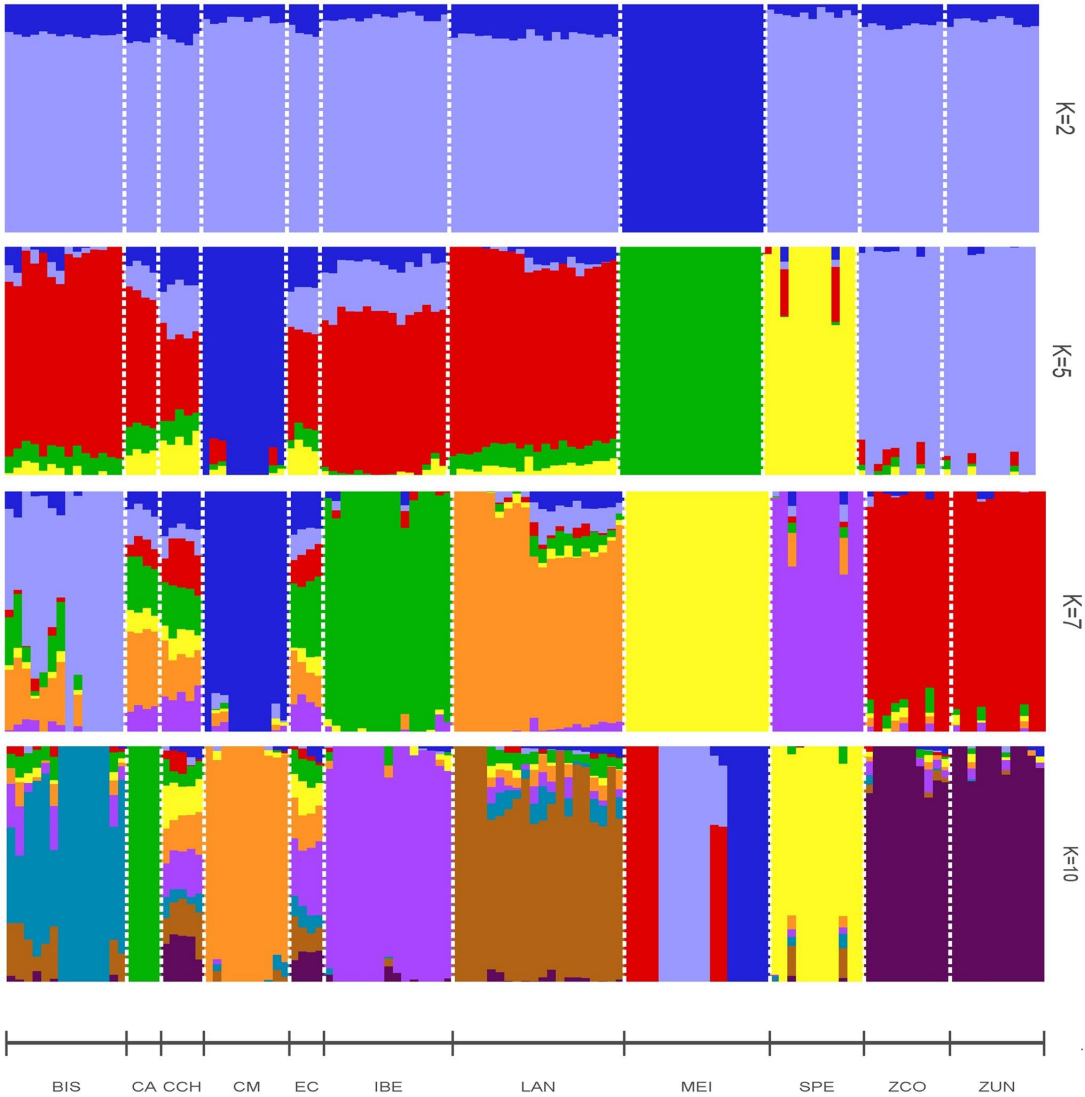
Clustering of pig populations, including colombian creole pigs based on admixture estimation for k = 2, 5, 7, and 10 hypothetical ancestral populations. The cross-validation error predicted the best k = 7. Each bar indicated an individual, and each color revealed the membership to a particular cluster

The admixture analysis indicated that the optimal model was k = 7 based on the cross-validation error (see Supplementary Fig. 1C). Figure 5 illustrates diverse k values, yet k = 2 produces two main clusters separating MEI from other populations. When k = 5, various clusters distinguished by their colors included ZUN, SPE, MEI, and CM. The remaining breeds were grouped in a singular color cluster. At the best k = 7, a cluster is formed in the ZUN, SPE, MEI, LAN, IBE, CM, and BIS animals (with some admixture). The CCH, EC, and CA populations resemble admixture groups of the other clusters. Finally, at k = 10, CA forms its separate cluster, but EC and CCH remain admixture groups.

**Fig. 6:**
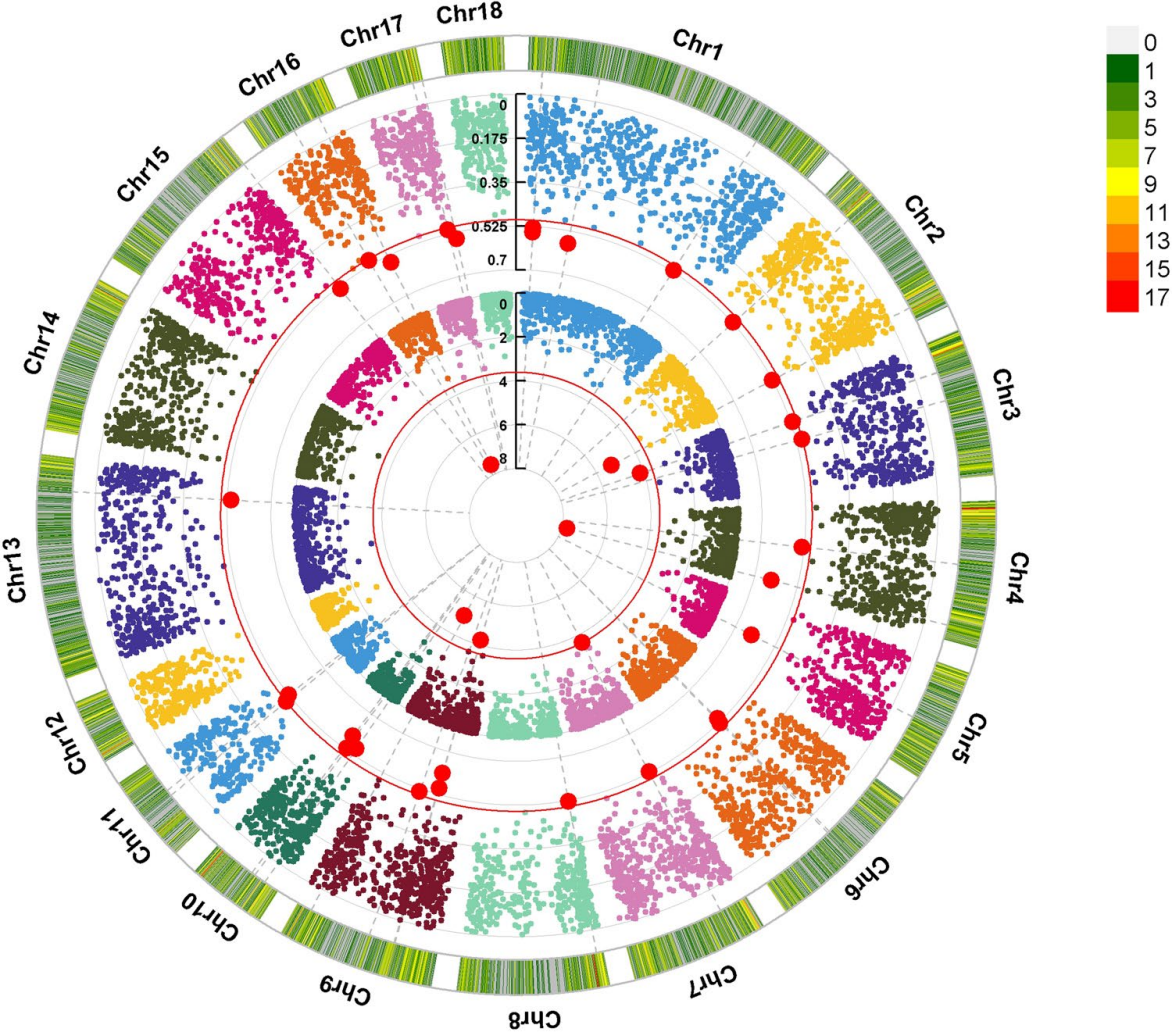
Circular Manhattan plot showing the SNP density per chromosome. The -log(p) values estimated with pcadapt (inside) and F_ST_ (outside) per SNP

Subsequently, to identify regions of the genome with a possible effect on environmental adaptation, an analysis of pcadadpt and F_ST_ by SNP was performed. The results are presented in a circular Manhattan plot (see Fig. 6). Pcadapt detected seven significant signals closely related to high F_ST_ peaks (F_ST_ greater than 0.5). We found common and significant signals on chromosomes 2, 3, 7, 9, and 16. Other relevant signals in the F_ST_ analysis were close to significance in pcadapt; however, these signals were not considered for further analysis.

Finally, the windows identified 113 genes from the signals, with 66 annotated in *Sus scrofa*. The genes included HS2ST1, SLC37A4, and U6 (Table 5), among others, related to important metabolic and immunological functions. For example, 19 genes were found related to negative regulation of biological processes (GO0048519), 17 with transport (GO0006810), eight with chemical homeostasis (GO0048878),, six with biological processes involved in symbiotic interaction (parasitism) (GO0044403), three with regulation of response to biotic stimuli (GO0002831), three with negative regulation of response to viruses (GO0050687). Despite this, the analysis found no reports of QTLs previously reported in the windows defined according to the AnimalQTLdb database.

**Table 5:** SNP name, chromosome (chr), position, and genes related to the structuring and differentiation of Colombian creole pigs

SNP Name	Chr	Position	Gene
H3GA0014365	4	128,626,976	HS2ST1
ALGA0091167	16	67,321,433	SGCD
MARC0105379	2	146,620,271	PRELID2
H3GA0028334	9	137,862,578	-
ASGA0104824	9	46,356,452	HINFP, ABCG4, THY1, NLRX1, CCDC153, NHERF4, CBL, MCAM, C1QTNF5, MFRP, USP2, NECTIN1, IFT46, ARCN1, PHLDB1, TREH, DDX6, CXCR5, BCL9L, UPK2, FOXR1, CENATAC, RPS25, TRAPPC4, SLC37A4, HYOU1, VPS11, HMBS, DPAGT1, C2CD2L, RNF26, H2AX
ALGA0114914	3	32,478,846	CLEC16A, TEKT5, EMP2, ATF7IP2, GRIN2A, CIITA, TVP23A, NUBP1, DEXI, U6
ASGA0032164	7	30,698,557	GRM4, PACSIN1, SPDEF, ILRUN, SNRPC, BLTP3A, TAF11, ANKS1A, TCP11, SCUBE3, ZNF76, DEF6, U6, HMGA1, SMIM29, RPS10, NUDT3

## Discussion

### Diversity, linkage disequilibrium, and effective population size

Colombian Creole pig breeds are an important genetic resource for the resilience of livestock production systems in tropical conditions. They are a production alternative for small farmers and represent a significant genetic pool for investigating alternative production methods in extreme weather scenarios, such as those anticipated. Despite this, as raised by DAD-IS (FAO 2019), the inventory of locally adapted breeds in Colombia is drastically limited. Reports show population censuses of less than 138 purebred animals for each of the official breeds, with the most critical situation being for SPE, with only 99 animals reported, indicating a high risk of genetic erosion. The above justifies the conduct of research studies that evaluate the conservation nucleus. This will help us better understand the diversity and genetic relationships with other breeds or biotypes to make informed decisions about the conservation, maintenance, and use of this zoogenetic resource, which is vital for tropical production. It is worth noting that this work has a relatively small sample due to the size of the population census and the official breeds’ conservation nucleus. Nonetheless, the sample is representative of the diversity within each breed, as it includes individuals from various family groups (approximately 10% of the population census for each breed).

In Colombia, several officially accepted breeds of indigenous pigs have also been reported to the Domestic Animal Diversity Information System (DAD-IS), such as CM, SPE, and ZUN (FAO 2019). Other populations are officially classified as Colombian Creole pig breeds, although unofficially, they are considered different breeds in some regions of Colombia. However, insufficient studies have been conducted to classify this genetic resource adequately. The CCH is one of these populations that is considered a distinct breed, close to the SPE pig (Oslinger et al. 2006) due to its particular breeding conditions: its location in the Pacific region of Chocó (jungle, without large pig production), difficult access, a humid tropical climate (one of the rainiest regions in the world), and a local breeding system in semi-feral conditions, where the animals live from scavenging and with little control over reproduction. Currently, there are no reports on the size of the existing population. However, it is believed to be much smaller than in the other Colombian breeds, with the worrying fact that there is no conservation nucleus because it is not characterized or officially classified as a breed. As mentioned above, sampling CCH animals was difficult due to the conditions in which the animals were found. In addition, in many areas where the CCH lives, animals from commercial lines of pigs bred in the same way have been found, suggesting the possibility of mixing, making it difficult to find phenotypically Creole pigs and limiting the likelihood of obtaining samples (hence the sample size).

The chip used showed many segregating markers in Colombian Criollo pigs, which is useful for assessing diversity. However, chips will always have a bias that makes it difficult to detect rare and unique variants that may be key to understanding evolutionary processes, in such studies it is advisable to work with whole genome sequencing data. In this study, the informativeness of the evaluated chip was high, with the lowest MAF values before pruning of 0.237 (CM and ZUN) and the highest of 0.276 in CCH. This value is high in all the breeds evaluated, especially in CCH. Some MAF values were similar but mostly lower than those reported in other European pig breeds, ranging from 0.133 in Turopolje to 0.294 in Sarda (Muñoz et al. 2019). Notably, genotyping arrays usually have an ascertainment bias due to the design of chip genotyping from some specific breeds with high use worldwide, so a local population as particular as CCH would not be expected to have the highest value. This could be related to introgression processes of breeds such as Landrace, Meishan, or synthetic lines (Large White and Pietrain), which are widely used in intensified production systems. However, it may also reflect efforts to produce increasingly informative chips like the one used in this study under the GeneSeek^®^ GGP Porcine80K platform. In any case, it has been previously shown that despite the ascertainment bias inherent in the chips, the results show their reliability for assessing genomic diversity (Herrero-Medrano et al. 2014), even in local breeds such as those in this study.

In addition, the mean LD among the Creole breeds was similar, with the lowest r^2^ value for Zungo (0.342), which also showed a faster decay of LD with distance (Fig. 1A), associated with greater genetic diversity. In contrast, despite the high MAF of CCH, its LD value was the highest, showing the highest LD persistence with distance. These higher values of LD persistence suggest that the breeds have experienced significant bottleneck and genetic drift events (Ardlie et al. 2002), which agrees with the generally low Ne values obtained, with the highest values in Zungo (Ne = 19) and the lowest in CCH breed (Ne = 10). This is consistent with a greater risk of genetic erosion and risk of extinction in CCH, given its small productive niche and the absence of a conservation program. In Colombia, a Ne of 13 was reported from pedigree analysis in the SPE breed (Ocampo-Gallego et al. 2019) and 16 for Zungo (Ocampo-Gallego and Abuabara-Pérez 2021), values very close to those reported in this study (16 and 19, respectively). Besides, using the 60 K chip in pigs, LD values higher than those in this study have been reported (r^2^ between 0.39 and 0.55), although at a different mean distance (Zhang and Plastow 2011). Lower and higher values have also been reported in European local breeds, with ranges between 0.289 and 0.604 as a mean evaluation measure up to 2 Mb. It is important to note that LD analyses are susceptible to the distance evaluated, genotyping density, chip, and quality control, among other variables, so one must be careful with comparisons. Regarding Ne, the values obtained were similar to those reported in local pig breeds such as Turopolje (Ne = 10), Mora Romagnola (Ne = 15), Lithuanian indigenous Wattle (Ne = 19) but are much lower than those found in breeds such as Bisaro (Ne = 62), Iberian (Ne = 89) or the wild boar (Ne = 23) (Muñoz et al. 2019).

When analyzing the other diversity statistics, it should be noted that the only population with a deficit of heterozygotes was the CCH (FIS = 0.093), with a mean Ho of 0.325 and He of 0.399, which is consistent with the higher persistence of LD, a lower Ne, and the genetic drift effect previously mentioned, associated with possible genetic erosion in this population. The other Colombian breeds showed negative F_IS_ values (-0.115 to -0.175), indicating a tendency toward exogamy associated with higher diversity. This could be because these breeds are kept in a conservation nucleus, initially formed by different families maintained under a cyclical circular mating scheme to control inbreeding and maintain genetic diversity. In addition, a representative sample of the purebred groups present in the country was included in the foundation of the population, which is consistent with the excess of heterozygotes found in the breeds. Values of Ho ranging from 0.195 to 0.363 have been reported in European pig breeds, with He in the range of 0.187 to 0.382. These values of heterozygosity evaluated from SNPs are lower and, in some cases, similar to those found in this paper (Muñoz et al. 2019). The above shows the Colombian breeds’ vital heterozygosity and the conservation nucleus’s good maintenance.

Values between 9.97% and 14.93% were found concerning ROH and autozygosity. The lowest homozygous genome coverage was found in the ZUN breed, indicating greater diversity, in agreement with the other statistics discussed previously. In addition, in this breed, there is a greater contribution to homozygosity of the small ROH related to old inbreeding events. At the same time, the larger ROH covered less of the genome, which matches the negative F_IS_ values mentioned previously. A similar result was found in the CM and SPE breeds, which show higher autozygosity of short fragments, indicating ancient inbreeding. However, in the CCH population, a higher coverage of long fragments is observed, pointing to more recent inbreeding and consequent genetic erosion. Notably, the highest value of autozygosity (14.9%) was presented by the SPE breed, which also has the lowest population census (Table 1), so care should be taken in the breeding schemes of the conservation nucleus. Higher values of F_ROH_ than those found in this investigation have been reported (38.6 and 28.1%) in Nero Lucano pigs, with a greater tendency toward recent inbreeding (Di Gregorio et al. 2023).

Similarly, higher values have been reported in different commercial breeds in Italy, but with a lower contribution of recent inbreeding (Schiavo et al. 2020), as in this study. Similar values of F_ROH_ (13.3%) were reported in Laiwu Pigs (Fang et al. 2021), showing a higher effect of old inbreeding compared to our work. Furthermore, a study analyzing inbreeding by pedigree in the same SPE population found a higher percentage of inbred animals in older generations and a decrease in recent generations (Ocampo-Gallego et al. 2019), which is consistent with the results presented in this study.

### Multibreed analysis

This is the first genome-wide study that includes the three recognized pig breeds from Colombia and uses information from the germplasm bank. However, a 60 K chip-based analysis was previously performed by Burgos-Paz et al. (2013) to understand the history of the colonization of pigs in the Americas. From that study, some genomic data were taken for our research, such as those reported from the ZUN pig that we used as a merging control between databases. In that study, the authors concluded that American Creole pigs are a complex conglomerate with mainly European ancestry. Still, the IBE pig was not always the main genetic component, showing an essential contribution of international and Chinese breeds for forming American Creole pigs (Burgos-Paz et al. 2013). In our work, this was so for the CCH and EC breeds, which were observed as a mixed group with a closeness to the IBE pig but with the composition of other breeds. The situation was different for the CM, SPE, and ZUN breeds recognized in Colombia, which had their cluster separated from the other breeds, with some mixing events but showing breed imprinting.

Furthermore, with the best k = 7, each Colombian breed has its cluster, although this is consistent with the work of Burgos-Paz et al. (2013), which shows ZUN as an independent and well-defined cluster. A structure analysis including the Colombian Criollo and LAN breeds (Barrera et al. 2007) reported that each has its cluster, as found in this work, indicating imprinting for the CM, ZUN, and SPE breeds.

Our analysis found a greater population structuring according to F_ST_ when other breeds were included (0.138 vs. 0.190). However, the magnitude of the F_ST_ only including Creoles is substantial (0.138). This is observed in the PCA (Fig. 2, first three components), where there is a separation of the different populations of Colombian Creoles, with the closest proximity between CM and SPE. Also, according to the PCA plot, the CCH pig is in an intermediate position between the Creoles and the Iberian pig, with a critical proximity to the EC and CA pigs. Something similar is observed in the NJ tree, based on the genetic distances of Nei, again showed the closeness of CM-SPE and between CCH-EC, as well as the separation of ZUN in an independent group relatively distant from the others, which is in agreement with what was shown in the other analyses.

In a 2007 microsatellite study using the CM, ZUN, and SPE breeds, CM and ZUN Creole breeds were reported to be very close in a multivariate analysis (Barrera et al. 2007). In contrast, the same study estimated Nei genetic distances and found greater distances between CM and ZUN. At the same time, the shortest spans were between CM and SPE, consistent with those we reported in this paper. Another study performed in Creole pigs using Random Amplified Microsatellites (RAMs) found a lower unbiased Nei distance between CM-SPE and the highest between ZUN-SPE (Oslinger et al. 2006). Regarding Nei distance with IBE pig, the lowest reported by Barrera et al. (2007) was with SPE (0.438) and the highest with CM (0.865), while in our study the lowest Nei distance was with CCH (0.111), followed by ZUN (0.130), SPE (0.147) and CM (0.147); the same was observed in the other analyses where CCH was closer to the Iberian pig. In another study, Revidatti et al. (2021) evaluating the origin of the American Creole pig, reported that the shortest genetic distance of Nei of the American Creole pigs was with IBE (0.061) and the longest with MEI (0.628), similar to what was found in this investigation and supporting the thesis of the Iberian ancestry of the American Creole pigs.

The tree of genetic distances presented in a previously mentioned study places the SPE in the same cluster with breeds such as LAN and separated from CM and ZUN (Barrera et al. 2007), contrary to those reported in this work, where LAN is separated from SPE. However, in the Treemix analysis, a significant introgression of an ancient group of LAN into SPE was observed, which could correspond to the relationship reported by Barrera et al. (2007). Nonetheless, in our work, the evaluated SPE population has its cluster in the admixture analysis, showing a LAN cluster composition in only two of the individuals being assessed (Fig. 5), which could suggest that in some of the founding families of SPE, there was crossing with LAN material at some point in their history.

Another study evaluating microsatellites reported SPE in the same cluster with commercial pig populations, Iberian populations, and the Duroc breed. It also reported EC, ZUN, and Colombian Creole in a separate cluster (Revidatti et al. 2021). In another recent study performed with Colombian Creole pigs, where the genetic diversity of the SLA-DRB1 gene was compared, Nei genetic distances were estimated from the gene. Using the BIONJ algorithm, they found that Colombian Creole pigs formed an independent cluster with Duroc (Celis-Giraldo et al. 2021), with a similar order of relationships between the Colombian breeds to that found in this work. Although our work did not include the Duroc breed, a meaningful relationship was found between Colombian Creoles and other pigs, such as IBE and EC. In addition, the ML Treemix showed that CCH is closer to ZUN. However, ZUN is a breed originating from the Caribbean region of Colombia and CCH from the Pacific region, which reveals a difficult recent transit between the locations, so their similarity may be due to founder effects from related ancestral populations. Moreover, the Treemix analysis also identified MEI introgression on the branch between CCH and ZUN, which is reflected to a lesser extent in the admixture analysis.

Similarly to what was previously stated for CCH, it featured the lowest values of Slatkin F_ST_ paired with the other Creole pig populations (0.14 to 0.16). However, CCH showed lower values even with the EC pig (0.04) and with LAN (0.09), reinforcing the idea of an intermediate or crossbred population (Fig. 2). In addition, the Netview population structure analysis (Fig. 4A) showed a vital gene flow between CCH, EC, and CA, and even a structuring in the IBE population, with a group closer to CA and the group above. Moreover, contrary to what was previously stated, SPE and CM had one of the highest Slatkin F_ST_ values among the Creoles (0.27). This analysis shows a critical separation between the three official breeds of Colombia, supporting the definition of different breeds and their imprinting.

According to historical accounts, the conditions of the new world favored the proliferation of pigs, and some even became wild, reproducing to such an extent that they were trapped with the help of hunting dogs. It is believed that the pig originally arrived through the coast to the department of Córdoba, where the ZUN descendant of the Lampiña breed originated, but due to the phenotypic diversity it is believed that other pigs different from the Lampiño also arrived in the country (Barrera et al. 2007; Ocampo-Gallego et al. 2019), which could partly explain the significant population structuring and the genetic diversity found in this work within the Colombian breeds. It may also be related to the close clustering between the CCH and EC pig above other Colombian Creoles. In addition, it is known that the ZUN breed is a descendant of the first pigs established in Córdoba (Caribbean coast). Conversely, the CM breed was established in the eastern plains (Orinoquia), and SPE settled in the Andean region, especially in Antioquia; all far from each other and adapted to different climatic characteristics and difficult transit, especially at the time of the colonization, which suggests that it was unlikely for pigs from a common origin and location to arrive in all these places (Barrera et al. 2007). The Netview analysis also suggests that there may be structuring within the IBE population even in current generations (Fig. 4A). In addition, there are reports that other livestock, such as cattle, arrived from the Caribbean coast. However, other groups from present-day Ecuador and Peru came to the country from the south (Barrera et al. 2007). This may also explain the relationship between CCH and EC, assuming that a similar migration process occurred in pigs.

This study reported signals in some regions of the genome associated with the negative regulation of biological processes, such as chemical homeostasis response to viruses and parasites. These signals were found mainly on chromosomes 2, 3, 4, 7, 9, and 16, with many genes associated with immune function. This is consistent with what was reported by Cross et al. (2018), who suggested that thermotolerance as an adaptative trait is heritable and proposed some candidate genes and pathways associated with metabolic and immunological functions, which were mainly located on six chromosomes, including 2 and 7. In addition, a study evaluating selection signals associated with heat tolerance in indigenous pigs found genes related to functions such as heat stress injury and anti-inflammatory, sperm development, pregnancy, and ovarian and mammary gland development, among others (Zhong et al. 2023), mainly located on chromosome 1, 3, and 6. Another study in Tibetan pigs looking for signs of selection to altitude reported genes related to angiogenesis, pulmonary hypertension, erythropoiesis, oxygen intake, and defense response (Ai et al. 2014). Genes associated with the defense response were also found in our study. The most important immune response genes were ILRUN, RNF26, NLRX1, TCP11, NECTIN1 and DDX6, but other genes related to homeostasis such as CXCR5, SGCD, GRIN2A, SLC37A4, NUBP1 and ABCG4 were also found (Table 5). In addition to particular genes related to response to light stimuli such as THY1, MFRP and NECTIN1. We only looked for genes associated with structuring among the breeds sampled in the present investigation. Still, the signals detected cannot be attributed to adaptation to a particular climatic condition. Therefore, it is recommended that future studies of selection signals associated with specific adaptive processes, such as adaptation to altitude, heat, reproductive performance, and disease resistance, among others, be carried out using different methods of population comparison and genomic association.

Finally, it is advisable to research the genetic and phenotypic characterization of the Colombian Creole biotypes, involving as many individuals as possible, to understand the genetic diversity present and thus facilitate the establishment of conservation nuclei. It is also relevant to make progress in the morphological and type characterization because, for example, in the CCH, the coat color is very heterogeneous, which could be a consequence of the introgression of other breeds. It is also important to conduct studies aimed at understanding the mechanisms of tropical adaptation, the signals of adaptation, and their relationship with the landscape to contribute to the understanding of the genes involved and the genetic architecture of this trait, which is vital for the resilience and productive future of tropical livestock production systems.

## Supplementary Information

Below is the link to the electronic supplementary material.

Supplementary file1 (DOCX 85.7 KB)

## Data Availability

The data sets generated or analyzed during the current study, or both, are available from the corresponding author upon reasonable request.
