# Supplementary material for: Genome-wide diversity, linkage disequilibrium, and admixture in the main Colombian Creole pig breeds
Source: Trop Anim Health Prod. 2024 Oct 10;56(8):336. doi: 10.1007/s11250-024-04140-6 (PMC11464582; doi:10.1007/s11250-024-04140-6)

**Supplementary 1.** Best values for clustering according to the methodology. A) Best maximum number of nearest neighbours (K_NN) for netview analysis. B) Best optimal number of migratory events between populations (m = 1–10) for the Treemix analysis. C) Plot of cross-validation error for k (k = 1–15) hypothetical clusters for the admixture analysis.


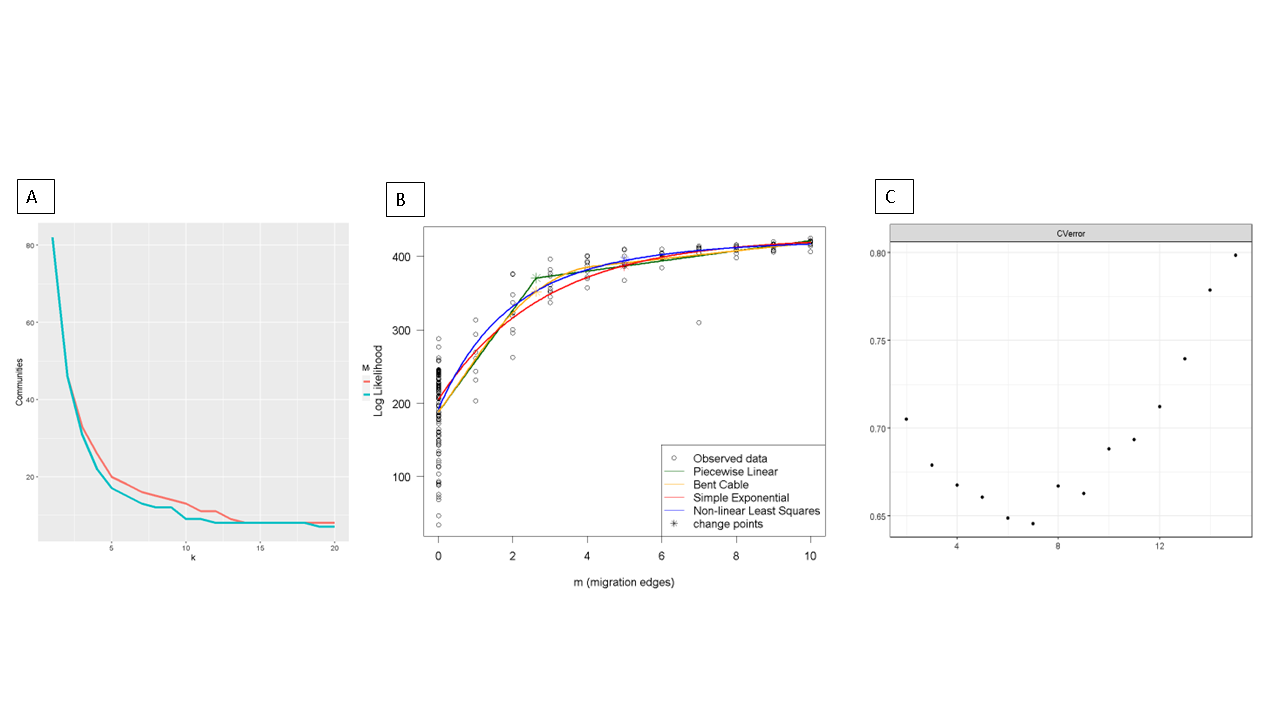

Supplement: Supplementary file 1 — Supplementary file1 (DOCX 85.7 KB) [file 11250_2024_4140_MOESM1_ESM.docx]
